# RGD‐functionalised self‐assembling peptide hydrogel induces a proliferative profile in human osteoblasts in vitro

**DOI:** 10.1002/psc.3653

**Published:** 2024-09-27

**Authors:** Luis A. Castillo‐Díaz, Julie E. Gough, Aline F. Miller, Alberto Saiani

**Affiliations:** ^1^ School of Chemical Engineering & Manchester Institute of Biotechnology The University of Manchester Manchester UK; ^2^ Departamento de Medicina y Ciencias de la Salud, Facultad Interdisciplinaria de Ciencias Biológicas y de la Salud Universidad de Sonora Hermosillo Sonora Mexico; ^3^ School of Materials & Henry Royce Institute The University of Manchester Manchester UK; ^4^ Division of Pharmacy and Optometry & Manchester Institute of Biotechnology The University of Manchester Manchester UK

**Keywords:** calcium deposition, human osteoblasts, hydrogels, RGD, self‐assembling peptides

## Abstract

Self‐assembling peptide hydrogels (SAPHs) have been used in the past decade as reliable three‐dimensional (3D) synthetic scaffolds for the culture of a variety of mammalian cells in vitro. Thanks to their versatile physicochemical properties, they allow researchers to tailor the hydrogel properties, including stiffness and functionality to the targeted cells and cells' behaviour. One of the advantages of using SAPH scaffolds is the ease of functionalisation. In the present work, we discuss the effect that functionalising the FEFEFKFK (F, phenylalanine; K, lysine; and E, glutamic acid) hydrogel scaffold using the cell‐binding RGDS (fibronectin — R, arginine; G, glycine; D, aspartic acid; S, serine) epitope affects the material properties as well as the function of encapsulated human osteoblast cells. RGDS functionalisation resulted in cells adopting an elongated morphology, suggesting attachment and increased proliferation. While this led to higher cell viability, it also resulted in a decrease in extra‐cellular matrix (ECM) protein production as well as a decrease in calcium ion deposition, suggesting lower mineralisation capabilities. The work clearly shows that SAPHs are a flexible platform that allow the modification of scaffolds in a controlled manner to investigate cell–material interactions.

## Graphical abstract

The effects of functionalising the FEFEFKFK (F, phenylalanine; K, lysine; and E, glutamic acid) hydrogel scaffold using the cell‐binding RGDS (fibronectin — R, arginine; G, glycine; D, aspartic acid; S, serine) epitope on the material properties and the function of encapsulated human osteoblast cells were studied. RGDS functionalisation resulted in an elongated cell morphology, suggesting attachment and increased proliferation, and decreased calcium ion deposition, suggesting lower mineralisation capabilities.

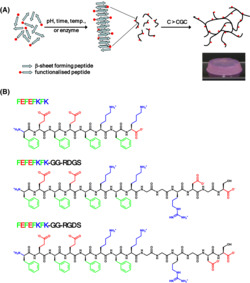

## 1 INTRODUCTION

Self‐assembling peptide hydrogels (SAPHs) have been used in the past decade as reliable three‐dimensional (3D) synthetic scaffolds for the culture of a variety of eukaryotic cells in vitro as well as vehicles for drug and cell delivery for tissue engineering and therapeutical applications in vivo.^1^, ^2^, ^3^ Thanks to their versatile physicochemical properties, they allow researchers to tailor the hydrogel properties, including stiffness and functionality, to the targeted cells and cells' behaviour.^4^, ^5^ SAPHs have therefore the potential to be fully defined alternatives to currently used animal‐derived scaffolds such as Engelbreth–Holm–Swarm (EHS)‐based matrices.^6^, ^7^, ^8^

SAPHs are formed through the self‐assembly of short peptides, typically four to 20 residues long, via non‐covalent interactions into nano‐fibrillar assembly and networks that mimic the extra‐cellular matrix (ECM) architecture. The use of natural peptides allows the formulation of biocompatible, non‐immunogenic, and biodegradable scaffolds that can potentially be translated to the clinic.^9^, ^10^, ^11^ One major advantage of SAPHs is their ability to mimic the mechanical properties of various soft tissues by either tuning the type of residues used to design the peptide primary sequence, varying the peptide concentration and composition or combining them with others polymeric materials.^12^, ^13^, ^14^

Bone repair is a particular active field as in addition to bone related trauma, the prevalence in the general population of bone related diseases such as osteoporosis, Paget's disease, bone cancer and periodontitis continue to increase.^15^ Various types of stems^16^, ^17^ and bone‐forming^18^, ^19^, ^20^ cells have been cultured in SAPHs in 3D in vitro. We have shown in the past that self‐assembling β‐sheet forming octapeptide FEFEFKFK (F, phenylalanine; K, lysine; and E, glutamic acid) hydrogels could be used for the 3D culture of human osteoblasts and allow, in the presence of osteogenic media, mineralisation and deposition of calcium phosphate ions.^19^ We then also showed that using differentiation media, human mesenchymal stem cells could be differentiated into osteoblasts in these hydrogels that once again were able to deposit calcium phosphate ions.^16^

**FIGURE 1:**
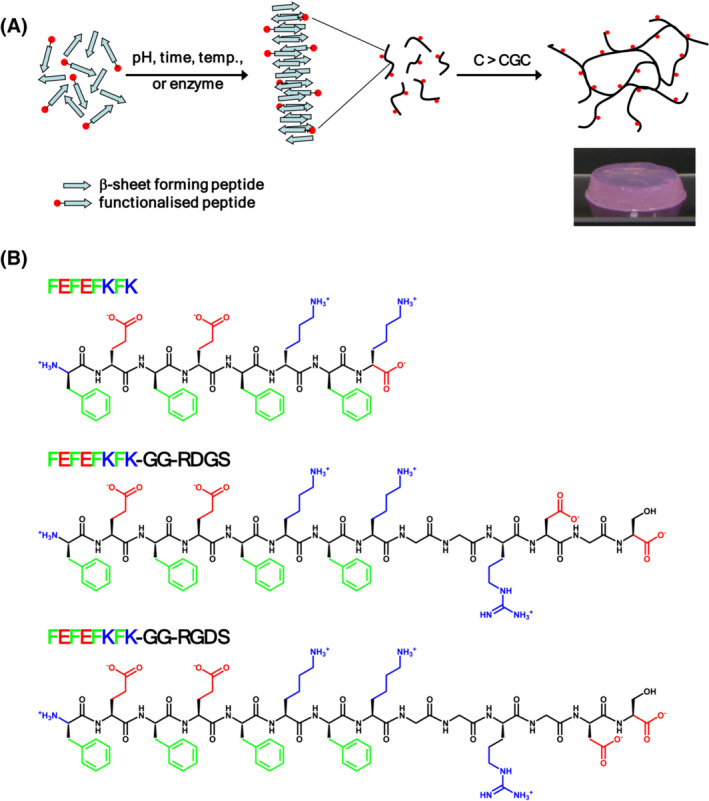
(A) Schematic representation of the self‐assembly and gelation process of β‐sheet forming self‐assembling peptides in hydrogels (SAPHs). To functionalise the hydrogels, peptide functionalised at their C or N‐termini with the desired peptidic epitope are introduced into the formulation. The photographs show a hydrogel obtained following conditioning with cell culture media. (B) Chemical structures of the peptides used in this work. CGC, critical gelation concentration.

One of the advantages of using β‐sheet‐forming SAPH scaffolds is the ease of functionalisation. Indeed, functional epitopes can be added to the end of the peptides and the β‐sheet self‐assembly process ensures that these epitopes are present on the surface of the fibres as shown schematically in Figure 1A.^21^ Indeed, we have functionalised in the past these peptides with a range of functional groups including peptidic biofunctional moieties,^22^ polymers,^23^, ^24^ enzymes^25^ and DNA fragments.^26^, ^27^

Some commonly used minimalistic short bioactive peptidic epitopes used to functionalise SAPHs include the following: RGDS (fibronectin — R, arginine; G, glycine; D, aspartic acid; S, serine), GFOGER (collagen — O, hydroxyproline), IKVAV (laminin — I, isoleucine; V, valine; A, alanine) and YIGSR (laminin — Y, tyrosine). These ligands are thought to be involved in promoting several cellular functions such as viability, proliferation, migration and differentiation through recognition by cell membrane integrin receptors.^3^, ^28^, ^29^ RGD, in particular, has been used extensively to functionalise various synthetic and naturally derived scaffolds including polyethylene and alginate, to improve cell viability and promote cell proliferation.^30^, ^31^, ^32^, ^33^

In the present work, we decided to functionalise our FEFEFKFK hydrogel scaffold using the RGDS epitope. This was achieved by introducing in the formulation 20% (wt. %) of FEFEFKFK‐GG‐RGDS functionalised peptide. We then investigated the effect that functionalising the hydrogel had on human osteoblast (hOB) cell function. As a control we also formulated hydrogels using the scrambled sequence RDGS using the FEFEFKFK‐GG‐RDGS peptide (Figure 1B).

## 2 MATERIAL AND METHODS

### 2.1 Materials

The peptides FEFEFKFK (M_W_ = 1121 g mol^**−**1^), FEFEFKFK‐GG‐RDGS (M_W_ = 1650 g mol^**−**1^) and FEFEFKFK‐GG‐RDGS (M_W_ = 1650 g mol^**−**1^) were purchased as HCl salts with a purity of > 95% (TFA content < 0.1%) from Biomatik Corporation, Kitchener, Canada. The peptide purity was confirmed by high‐performance liquid chromatography (HPLC) and mass spectrometry (MS) by the supplier. All other chemicals were purchased from Sigma and used as received.

### 2.2 Hydrogel formulation

Hydrogels were prepared at a final peptide concentration of 30 mg mL^−1^. 36 mg of peptide powder was dissolved in 800 μL doubly distilled water and vortexed. To prepare the modified hydrogels, 20% by weight (7.2 mg) of the FEFEFKFK peptide powder was replaced by the modified peptide FEFEFKFK‐GG‐RDGS or FEFEFKFK‐GG‐RGDS. The peptide solutions were then centrifuged (4000 rpm min^−1^) and incubated at 90°C for 2 h to ensure complete peptide dissolution. The gelation was induced through stepwise addition of 105 μL of a concentrated NaOH (1 M) solution and 100 μL of Dulbecco's phosphate buffer saline (DPBS) solution. Finally, the hydrogels were placed under UV light for 15 min to ensure sterility and then stored at 4°C for at least 12 h before use. For additional information of the formulation of these hydrogels, please see reference [19].

### 2.3 Transmission electron microscopy (TEM)

Samples were prepared at 10 mg mL^−1^ and diluted 10‐fold. The solutions were vortexed until they were fully homogeneous. A total of 20 μL of sample was adsorbed onto the glow‐discharged, carbon‐coated copper grid (400 mesh, Agar Scientific) for 30 s. The loaded grids were washed in distilled water for 15 s and negatively stained with 20 μL of 1% (w/v) uranyl acetate. The grids were then blotted on Whatman 50 filter paper and allowed to air dry for 30 min prior to observation. Data was collected at high vacuum on a FEI Tecnai12 BioTwin transmission electron microscope connected to a high‐resolution Orius CCDSC1000 camera.

### 2.4 Oscillatory shear rheometry

The shear moduli of the hydrogels in cell culture conditions with and without cells were measured using an ARG2 rheometer (TA Instrument) equipped with a 20‐mm parallel plate at days 1, 7 and 14. One hundred and fifty microlitres of gel was pipetted onto the bottom plate of the rheometer, and the top plate lowered and left for 10 min to equilibrate before measurement. A gap of 250 μm was used, and the elastic moduli, *G*′, were measured at 1 Hz and 1% strain within, for all materials, the linear viscoelastic region.^34^, ^35^ All samples' measurements were performed at physiological temperature, 37°C, using a Peltier stage and solvent trap to minimise water evaporation.

### 2.5 3D cell culture

Primary human osteoblasts (hOBs) were grown using standard growth cell culture media (PromoCell, Heidelberg, Germany) and maintained under standard incubation conditions (37°C, 5% CO_2_, saturated humidity). Once at 70% confluence, the hOB cells were retrieved from the culture flasks and cell suspension (50 μL) carefully transferred (1.5 × 10^6^ cells) onto the hydrogels (200 μL). The cell suspension on the surface on the hydrogels was then gently mixed into the hydrogels using the tip of a pipette (up and down pipetting) until a homogeneous cell dispersion was obtained. The cell laden hydrogels (250 μL) were then transferred onto 12‐well cell culture inserts (ThinCert™ Greiner Bio‐One). Fresh media was added into the cell culture well and on top of the hydrogels and samples incubated for 10 min. Five cell culture media changes (20 min intervals) were then carried out during the first hour. Subsequently, media changes were performed every 2 days over the 14‐day cell culture experiment.

### 2.6 Cell viability

hOB viability was estimated using a live or dead assay (Invitrogen, UK). DPBS (1.5 mL) containing 2.5 μL of 4 μM ethidium homodimer‐1 (EthD‐1) assay solution and 1.5 μL of 2 μM calcein AM assay solution were prepared. The live or dead assay solutions were pipetted on top of each hydrogel and then incubated under standard cell culture conditions for 20 min. The staining solution was then removed, and samples were viewed under a Leica TCS SP5 confocal microscope. For each sample, confocal Z‐scan series from five random areas were obtained at days 1, 7 and 14. Cell viability was calculated by dividing the number of live cells (green) by the total number of cells − live + dead (green + red).

### 2.7 Cell proliferation

hOB cell proliferation in the hydrogels was quantified using PicoGreen dsDNA assay (Life Technologies, UK). After the hydrogels were rinsed twice with DPBS, they were resuspended in 500 μL of cold lysis buffer (200 mM Tris–HCl, 20 mM ethylenediaminetetraacetic acid (EDTA)/double‐distilled water (ddH_2_O)/1% Triton X‐100) for 25 min. To ensure complete lysis, samples were vortexed vigorously and subject to three freeze–thaw cycles. Samples were then vortexed again and gel–cell suspension (100 μL) plated into black 96‐well plates, to which 100 μL of a working solution of Quant‐iT PicoGreen reagent was added. The samples were incubated for 2–5 min at room temperature (RT). Fluorescence readings were obtained using a plate reader (FLUOstar OPTIMA; BMG LABTECH). A standard curve was established using calf thymus DNA in serial dilutions in 1% Triton X. A blank gel was used to correct the background absorbance, and the assay was performed in triplicate.

### 2.8 Immunochemistry

After 24 h of culture, each sample was cultured in osteogenic media containing dexamethasone (10–5 mM) (D4902‐16; Sigma‐Aldrich Co.), β‐glycerophosphate (10 mM) (G9422‐100G; Sigma‐Aldrich Co.), and ascorbic acid (2.83 × 10^−7^mM) (A8960‐5G; Sigma‐Aldrich Co., St. Louis, MO, USA). At each time point, the hydrogels were then fixed with paraformaldehyde (3%) for 30 min at RT. Thereafter, samples were permeabilised using Triton X‐100 (0.05%) in DPBS for 15 min at RT. Samples were subsequently blocked with bovine serum albumin (BSA) (1%) for 40–50 min at RT and incubated for 1 h at RT with a primary antibody (pAb) rabbit polyclonal to col‐I (Abcam, UK) diluted in BSA (1%), at a ratio stipulated by the manufacturer (1: 250). Subsequently, the samples were incubated for 1 h at RT in the dark with a goat anti‐rabbit IgG‐Alexa Fluor 594 (Abcam) as secondary antibody (sAb) along with Alexa fluor 488 phalloidin to target col‐I and F‐actin, respectively. The samples were rinsed in cycles of 5 × 5–10 min, with DPBS washings between each step. Samples were mounted on glass slides using ProLong Antifade Reagent (Invitrogen), and images were obtained using a Leica TCS SP5 confocal microscope.

### 2.9 ECM protein quantification

At each time point, gels were rinsed twice with DPBS and resuspended in cold distilled water to ensure gel dissolution. Cells within these samples were subjected to three freeze–thaw cycles which led to cell lysis. Three markers were selected, and procedures are outlined below. In each case, a blank gel was used to correct the background absorbance.

### 2.10 Collagen quantification

After resuspending and lysing the cells, samples were vortexed and hydrolysed over 20 h at 95°C with 12 M HCl (100 μL). Samples were subsequently diluted 1:1 in 4 M HCl and pipetted into a 96‐well plate, before adding the total collagen assay QuickZyme buffer (75 μL) (Biosciences, Park, Leiden, The Netherlands) and incubating for 20 min at RT. Following this, a detection reagent (75 μL) was added, and the sample transferred into the oven (60°C) for 1 h. Absorbance measurements were obtained at 570 nm, using a plate reader (Tecan Infinite M200). Three independent assays were undertaken in triplicate.

### 2.11 Osteocalcin quantification

Osteocalcin (OCN) production was determined using a Human Osteocalcin ELISA Kit (Invitrogen–Life Technologies). After the cells were lysed, samples were vortexed and pipetted into a 96‐well plate. Subsequently, a working anti‐OST‐HRP solution (100 μL) was added to each well; the plate was covered and incubated for 2 h at RT. Each well was then rinsed three times with a washing solution before adding a chromogen solution (tetramethylbenzidine) (100 μL) and incubating for 30 min at RT in the dark. The reaction was stopped by adding a stop solution (1 N HCl) (100 μL). Absorbance measurements were obtained within 1 h at 450 nm, using a plate reader (Tecan Infinite M200). Two independent assays were performed in triplicate.

### 2.12 Alkaline phosphatase activity quantification

Alkaline phosphatase (alkphos) activity was monitored using a colorimetric alkphos and peroxidase substrate detection system (Sigma‐Aldrich Co.). Cell lysate (20 μL) was added to transparent 96‐well plates together with *p*‐nitrophenyl phosphate (pNPP) solution (200 μL) (1 mg mL^−1^ pNPP, 0.2 M Tris buffer in 5 mL ddH_2_O) (SIGMAFAST™ pNPP Tablets, N1891‐50SET; Sigma‐Aldrich Co.), and the following reaction was stopped with 3 M NaOH. The absorbance of samples was measured using a plate reader (Labsystems Multiskan Ascent; Thermo Scientific, UK) at 405 nm every 30 s for 30 min. Two independent assays were performed in triplicate.

### 2.13 Calcium ion deposition

Calcium ion deposition was evaluated using Alizarin Red staining. At days 7 and 14, the cells were fixed with 70% ethanol for 30 min, rinsed three times with ddH_2_O and then stained with 40 nM Alizarin Red at RT in the dark for 45 min. Samples were then rinsed eight times with DPBS. For optical density measurements, 10% cetylpyridinium chloride (CPC) was dissolved in 10 mM sodium phosphate and 1 mL of this was added to each gel before incubating at RT for 30 min. A volume of 200 μL of each solution was plated into a clear, flat‐bottomed 96‐well plate (Nunc, UK) and the absorbance at 562 nm determined using a Labsystems Multiskan Ascent plate reader. The assay was performed in triplicate.

### 2.14 Statistical analysis

For statistical analysis, GraphPad (Prism 5) software was used. Cell numbers, oscillatory rheology and protein expression data are presented as mean ± SD of individual groups from 2 to 3 independent experiments. Statistical comparison between groups were carried out using one‐way ANOVA followed by post hoc comparisons (Tukey's method). A value of *p* < 0.05 was considered significant.

## 3 RESULTS AND DISCUSSION

**FIGURE 2:**
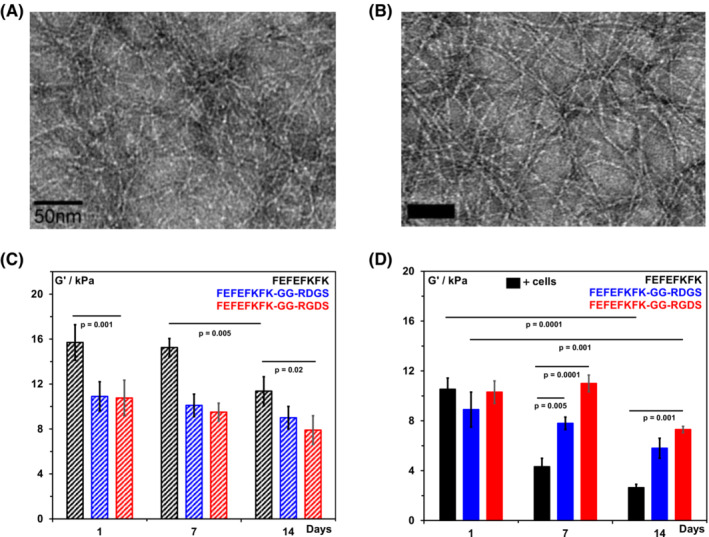
Top: transmission electron microscopy (TEM) images of FEFEFKFK (A) and FEFEFKFK‐GG‐RGD (B) β‐sheet fibres; bottom: storage shear moduli, *G*′, collected at 1 Hz and 1% strain of hydrogels (C) and cell‐laden hydrogels (D) at days 1, 7 and 14. For both sets of hydrogels, the same frequency of media changes and storage conditions was used over 14 days.

First, we investigated the effect that modifying the hydrogels had on their mechanical properties. In Figure 2A, the storage moduli, *G*′, obtained for all three hydrogels formulated at 30 mg mL^−1^ after cell culture media conditioning (day 1) are presented. As can be seen, FEFEFKFK formed a stiffer hydrogel with a *G*′ of ~ 16 kPa compared to the two modified hydrogels that had *G*′ of ~ 11 kPa. As discussed in our recent work, fibre–fibre interactions play a key role in determining the overall mechanical properties of these family of SAPHs.^34^, ^35^, ^36^, ^37^, ^38^ The introduction of GG‐RDGS and GG‐RGDS at the C‐termini will affect the nature of the interactions between the edges of the β‐sheet fibres, making them less hydrophobic in this case, resulting in the formation of weaker hydrogels. When hOB cells were encapsulated (Figure 2B), the *G*′ of the three cell‐laden hydrogels were found to be similar at day 1, ~ 10 kPa. As for all composite materials, the introduction of cells, which can be seen as micro‐fillers, will affect the materials bulk mechanical properties measured. The bulk *G*′ measured here will include the contribution of the cells' mechanical stiffness as well as the nature of the interface between the cells and fibrillar network.^39^, ^40^, ^41^

The hydrogels (with and without cells) were then subjected to the same experimental protocol as used for cell culture, that is, same media change frequency and storage in an incubator and the *G*′ measured at days 7 and 14 (Figure 2). The hydrogels without cells were found to be relatively stable over the 14 days with FEFEFKFK hydrogel showing the most marked decrease in *G*′ from ~ 16 kPa at day 1 to ~ 11 kPa at day 14. When cells were present, the decrease in *G*′ was significantly more marked going from ~ 10 to ~ 2 kPa over the 14 days suggesting that osteoblasts are degrading and remodelling the FEFEFKFK matrix. When the hydrogel was functionalised with GG‐RGDS, its *G*′ was found to be minimally affected by the presence of the cells suggesting that in this case, the hOB cells were not actively degrading and remodelling the matrix. Finally, for the hydrogel modified with the scrambled GG‐RDGS, a small decrease in *G*′ was observed suggesting an intermediate cell behaviour in this case.

**FIGURE 3:**
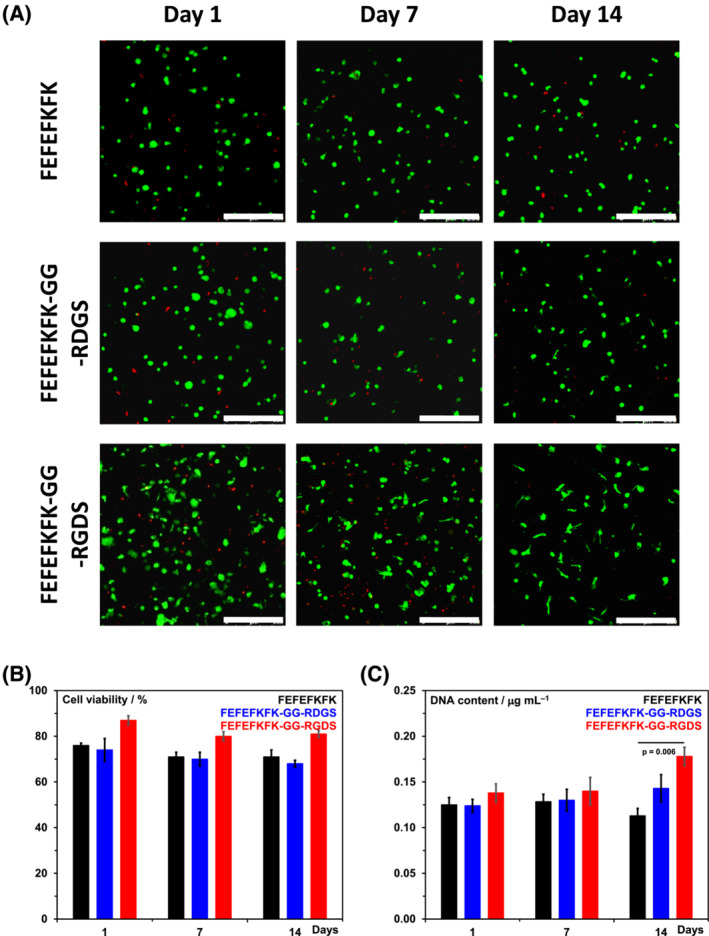
(A) Fluorescence microscopy images of live or dead stained cells at days 1, 7 and 14 obtained for hOB cells encapsulated in the three hydrogels — live cells are stained green and dead cell DNA is stained red. (B) Quantification of cell viability. (C) DNA content measured via PicoGreen assay.

Next, the hOB cell viability was investigated using a live or dead assay. In Figure 3A, the fluorescence microscopy images obtained at days 1, 7 and 14 are presented, and as can be seen, good cell viabilities in all three hydrogels were observed. This was confirmed via image analysis quantification (Figure 3B). For FEFEFKFK and FEFEFKFK‐GG‐RDGS hydrogels, cell viabilities of ~ 73% were obtained, while a slightly higher viability of ~ 85% was obtained for the FEFEFKFK‐GG‐RGDS functionalised hydrogel. Interestingly, the morphology of the hOB cells in the GG‐RGDS functionalised hydrogel was found to become elongated, spindle‐like, over time, suggesting that cell attachment occurred in this scaffold (Figure 3A). On the other hand, in FEFEFKFK, hydrogel cells were found to keep their round morphology suggesting in this case a lack of attachment. As above in this case too, the hydrogel modified with the scramble sequence, GG‐RDGS, showed an in‐between behaviour with a small fraction of cells presenting an extended morphology. This is thought to be due to some level of unspecific recognition by the cells of the scramble sequence RDGS occurring. Indeed, it has been reported in the literature that other modified sequences such as RADSP (original sequence RGDSP) as well as GRGESP and GRADSP (original sequence GRGDSP), can lead to cell showing equivalent behaviour to the non‐scrambled sequence albeit usually at a significantly reduced level.^32^, ^42^, ^43^, ^44^

We also investigated cell proliferation by measuring the quantity of DNA present in each sample over the 14 days of culture. As can be seen, from Figure 3C, the amount of DNA and therefore the cell number was found to significantly increase at day 14 for the GG‐RGDS functionalised hydrogel, pointing towards cell proliferation occurring in this hydrogel. On the other hand, for the non‐functionalised FEFEFKFK hydrogel, the amount of DNA present was found to remain relatively constant over the 14 days of culture with a small decrease observed at day 14. The hydrogel modified with the scrambled GG‐RDGS sequence once more showed an in‐between behaviour with a slight increase in DNA content being observed at day 14.

**FIGURE 4:**
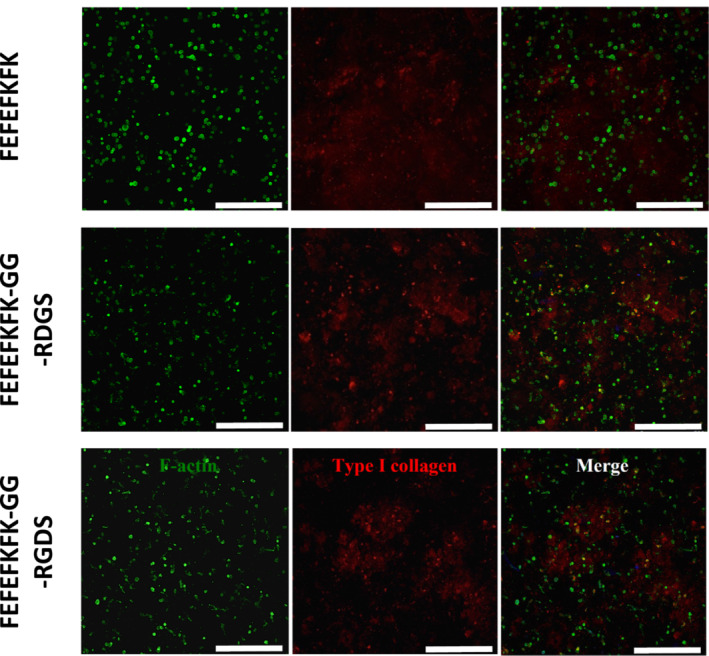
Fluorescence microscopy images obtained at day 14 following hydrogels stained for F‐actin (green — left) and collagen I (red — middle). Right column shows merged images.

**FIGURE 5:**
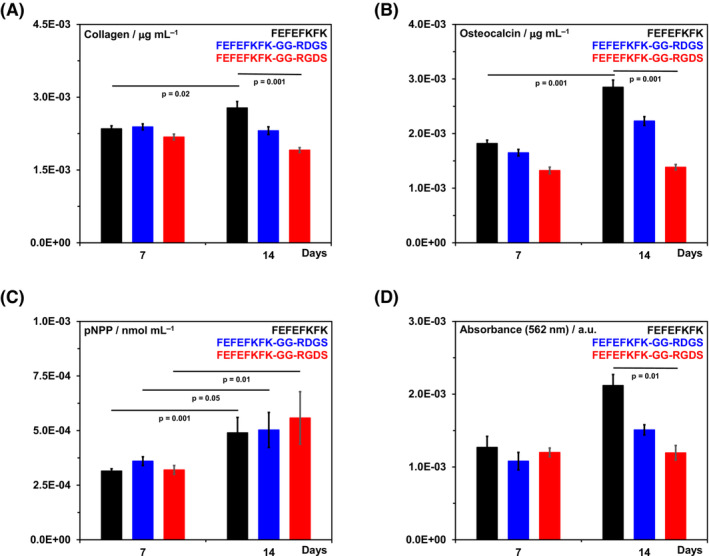
(A, B) Total collagen and osteocalcin present in the samples at days 7 and 14 respectively. (C) pNPP activity measured at days 7 and 14. (D) Alizarin red normalised absorbance as a measure of the quantity of inorganic calcium ions deposited in each sample.

Finally, we investigated the effect that functionalising the hydrogel had on protein production and calcium ions deposition. Collagen I is the most prominent protein found in the bone ECM and typically supports the formation of osteoid (non‐mineralised bone tissue), which is subsequently mineralised through calcium deposition by osteoblasts.^45^ In Figure 4, the fluorescent microscopy images of the sample at day 14 after staining for F‐actin (cell cytoskeleton) and collagen I are presented. As can be seen, extra‐cellular collagen I is produced by the hOB cells in all three hydrogels. Quantification of the total collagen produced at days 7 and 14 showed an increase in collagens deposition in the non‐functionalised FEFEFKFK hydrogel (Figure 5A). The two other modified hydrogels showed no significant changes in the amount of collagen present at days 7 and 14 with the GG‐RGDS functionalised hydrogel showing a small decrease. Similarly, the amount of osteocalcin, a calcium‐binding protein produced by osteoblasts and supporting mineralisation, was also found to increase significantly from days 7 to 14 in the non‐functionalised FEFEFKFK hydrogel (Figure 5B). In this case, a smaller increase in the amount of osteocalcin detected between days 7 and 14 was also found for the GG‐RDGS‐modified hydrogel, while no change was observed for the functionalised GG‐RGDS hydrogel. Next, the activity of alkaline phosphatase, an enzyme that is involved in calcification and formation of bone, was measured. Similar increases in pNPP activities were observed in all three hydrogels from days 7 to 14 (Figure 5C).

The protein expression profile found for the three hydrogels suggest that increased mineralisation is promoted by non‐functionalised FEFEFKFK hydrogels. This was confirmed by measuring the amount of inorganic calcium ions deposited in the three hydrogels at days 7 and 14 using alizarin red staining. As can be seen from Figure 5D, an increased amount of calcium ion deposition was observed for the FEFEFKFK hydrogel from days 7 to 14. For the GG‐RGDS‐functionalised hydrogel, no change in calcium deposited was observed, while for the hydrogel modified with the scramble sequence, GG‐RDGS, only a small increase in calcium deposition was seen.

Other studies have indeed also shown that RGD functionalisation can lead to cell attachment and proliferation being promoted, while bone protein formation decreases. For example, Benoit et al. have shown for murine osteoblasts a similar effect as observed here when functionalising their polyethylene scaffolds with RGD.^46^ It is thought that RGD functionalisation of 3D scaffolds triggers the formation of cytoskeletal stress fibres related to cell attachment and proliferation, which in turns downregulates the production of ECM proteins.^47^, ^48^, ^49^

## 4 CONCLUSION

We have investigated the effect that functionalising FEFEFKFK SAPH with RDGS cell adhesion epitope had on the hydrogel properties and hOB cell function. The functionalisation resulted in the cells adopting elongated morphology suggesting attachment to the scaffold and increased proliferation. While this led to higher cell viability, it also resulted in a decrease in ECM protein production, as well as a decrease in calcium ions deposition, suggesting lower mineralisation capabilities. The work clearly shows that SAPHs can act as a flexible platform that allows to modify scaffolds in a controlled manner to investigate cell–material interactions. Introducing bioactive peptidic epitope can clearly direct cell function but care needs to be used in the choice of epitope depending on the exact cell function targeted.

It should also be kept in mind that a range of factors will influence cell response to biological functionalisation of scaffolds; these include concentration, exact sequence and physical presentation. In addition, the introduction of functional motive can also influence the physicochemical properties of scaffolds, such as fibre surface charge and bulk mechanical properties that in turn can also affect cell response. Elucidating how all these parameters affect cell behaviour is critical to the design of novel synthetic functional scaffolds that can direct cell behaviour and fate.
